# Adolescents’ Experiences and Their Suggestions for HIV Serostatus Disclosure in Zambia: A Mixed-Methods Study

**DOI:** 10.3389/fpubh.2017.00326

**Published:** 2017-12-15

**Authors:** Sumiyo Okawa, Sylvia Mwanza-Kabaghe, Mwiya Mwiya, Kimiyo Kikuchi, Masamine Jimba, Chipepo Kankasa, Naoko Ishikawa

**Affiliations:** ^1^Department of Community and Global Health, Graduate School of Medicine, The University of Tokyo, Tokyo, Japan; ^2^Department of Educational Psychology, Sociology, and Special Education, School of Education, University of Zambia, Lusaka, Zambia; ^3^Pediatric HIV Centre of Excellence, University Teaching Hospital, Lusaka, Zambia; ^4^Institute of Decision Science for a Sustainable Society, Kyushu University, Fukuoka, Japan; ^5^Bureau of International Health Cooperation, National Center for Global Health and Medicine, Tokyo, Japan

**Keywords:** HIV, disclosure, adolescent, Zambia, mixed-methods study

## Abstract

**Background:**

HIV serostatus disclosure is an immense challenge for adolescents living with HIV, their caregivers, and health workers. In Zambia, however, little guidance is available from the adolescents’ point of view on the HIV disclosure process.

**Objective:**

This study aimed to examine the setting of HIV serostatus disclosure for adolescents, its impacts on them, and their suggestions on the best practice of HIV disclosure.

**Methods:**

We conducted a mixed-methods study at the University Teaching Hospital in Zambia from April to July 2014. We recruited 200 adolescents living with HIV, aged 15–19 years. We collected data using a structured questionnaire including two open-ended questions. We excluded two adolescents due to withdrawal during the survey, and eight from the data set due to out-of-eligibility criteria in age. Eventually, we included 190 in the analysis. We performed descriptive analysis to calculate the distributions of basic characteristics of the adolescents, their experience and preference on HIV serostatus disclosure, its emotional and behavioral impacts, and health education topics they had ever learned at hospital. We performed thematic analysis with open-ended data to explain first impressions upon disclosure in detail and to determine perceived advantages of HIV serostatus disclosure.

**Results:**

The majority of adolescents recommended the age of 12 as appropriate for adolescents to learn about their HIV serostatus and preferred disclosure by both parents. Out of 190 adolescents, 73.2% had negative or mixed feelings about HIV serostatus disclosure, while 86.2% reported that disclosure was beneficial. Thematic analyses showed that the adolescents reacted emotionally due to an unexpected disclosure and a belief of imminent death from HIV. However, they improved adherence to treatment (84.7%), limited self-disclosure of their HIV serostatus to others (81.1%), and felt more comfortable in talking about HIV with their caregivers (54.2%). Thematic analysis identified perceived benefits of disclosure as follows: better understanding of their sickness and treatment, and improved self-care and treatment adherence. Lower percentage of the adolescents have learned about psychosocial well-being, compared to facts about HIV and treatment.

**Conclusion:**

Despite initial emotional distress experienced after the disclosure, knowing one’s own HIV serostatus was found to be a crucial turning point for adolescents to improve motivation for self-care. HIV serostatus disclosure to adolescents requires follow-up support involving parents/primary caregivers, health workers, and peers.

## Introduction

Improved access to HIV testing and antiretroviral therapy (ART) has reduced the number of deaths caused by HIV (1). However, the adolescent population had not made the same progress by 2013 (2). Globally 250,000 adolescents aged 10–19 years were newly infected with HIV in 2015, and 41,000 lost their lives in the same year (3). Diagnosis of HIV infection and taking ART with good adherence are the principal means of suppressing viral load and maintaining a healthy condition (4, 5). However, adherence to ART is a great challenge among adolescents. A meta-analysis of 53 countries reported that only 60.1% of adolescents were adherent to ART in various measurements of adherence (6).

Adherence to ART among adolescents is affected by multiple factors including family structure, psychosocial and socioeconomic characteristics, treatment regimen, and access to health-care services (7, 8). Even after initiating ART, adolescents do not necessarily know their HIV serostatus and the actual reasons for taking medicines daily, and such knowledge gaps are critical barriers to ART adherence (8, 9). After adolescents learn their HIV serostatus, they commonly improve adherence to ART (10, 11), which could contribute to delayed disease progression and death (12). This highlights the importance of disclosing HIV serostatus to adolescents.

Disclosing HIV serostatus to adolescents impacts on self-care behavior and psychosocial well-being. For example, adolescents who were told about their HIV serostatus were more likely to retain in care (13) and were able to receive social support (11, 14–16). On the other hand, disclosing HIV serostatus to adolescents could generate complex feelings. Commonly, they are strongly distressed when they are informed about their HIV serostatus, while some get a feeling of relief (17). Acceptance of HIV serostatus would not be an easy process. However, adolescents desire to be given correct information on their HIV serostatus, and the purpose of taking medicine and having regular check-ups at the hospital (11, 18), and caregivers believe that adolescents should be told about their status (19, 20).

Disclosing HIV serostatus to adolescents is a difficult task for caregivers and health workers. Perceived barriers of disclosure for the caregivers and health workers are inadequate maturity and coping skills of adolescents; fear of inflicting emotional distress on them; fear of being blamed by them; potential risk of the HIV serostatus disclosed outside of their households with subsequent risk of social exclusion (19, 21, 22). In addition, caregivers and health workers do not have sufficient skills to disclose as they rarely have opportunities for learning and training about how to disclose HIV serostatus to adolescents (22–24).

Several studies have examined how serostatus disclosure is practiced (15, 25, 26), and in 2011, the World Health Organization published “Guideline on HIV disclosure counselling for children up to 12 years of age” (27). The guideline recommends that those aged 12 years or younger should be informed about their HIV serostatus, taking into account their maturity and capacity to understand (27). It would be beneficial to update the guidelines and include recommendations for adolescents reflecting more of their experiences and preferences. However, the majority of previous studies targeted caregivers and health workers as study participants; adolescents were less involved. It would also be important to reflect socio-cultural specificity, so that the guidelines could be adopted effectively in clinical practice.

Zambia is one of the highest HIV-burdened countries, with an estimated prevalence of 12.9% among adults in 2015 (3). About 68,000 adolescents were living with HIV, and 10% of them were newly infected (3). Coverage of ART expanded its reach to 63% of people in need in 2015 (3). However, adolescents’ HIV-related knowledge is still limited. The Demographic and Health Survey 2013–2014 reported only 42.3% of male adolescents (aged 15–19 years) and 38.9% of female adolescents had correct knowledge about transmission and prevention of HIV (28). This implies that adolescents living with HIV would be vulnerable to stigma and discrimination in an environment where people have inaccurate knowledge and negative views about HIV (29). Using qualitative methods, previous studies examined caregivers’ motivations and concerns as well as the process and impacts of HIV serostatus disclosure to adolescents (14, 22, 30). Two quantitative studies assessed mental health issues (31) and adherence to ART (32) and reported that serostatus disclosure was one of the factors associated with these outcomes.

However, no study has yet to quantitatively assess HIV serostatus disclosure to adolescents in Zambia. This study aimed to examine the actual setting and impact of HIV serostatus disclosure to adolescents, and their suggestions on the best practice of HIV disclosure.

## Materials and Methods

### Study Setting

We conducted a mixed-methods study at the Pediatric Centre of Excellence (PCOE), and Adult HIV Centre of Excellence (ACOE), University Teaching Hospital in Lusaka from April to July 2014. PCOE and ACOE are the national referral centers for HIV treatment and the national model institutions for the adolescent HIV care and treatment program. PCOE provides services to children and adolescents below 16 years, while ACOE is in charge of older patients including those transferred from PCOE.

### Participants

We recruited a non-randomized sample of adolescents living with HIV. The eligibility criteria for the study participation were as follows: the age of 15–19 years, regularly attending treatment at PCOE or ACOE every 3 months, and awareness of their HIV-positive status before the survey. At PCOE, parents/caregivers and health workers are encouraged to disclose HIV serostatus to adolescents beginning at the age of 10, according to their maturity and cognitive development. Thus, it was very rare for someone in the target group not to be aware of his/her HIV serostatus at the time of recruitment. Adolescents visit the centers for clinical review every 3 months. Thus, we had a 3-month survey period to recruit maximum number of participants. Recruitment was done during their waiting time for clinical review at the PCOE or ACOE premises. Before recruiting an adolescent into the study, we confirmed the adolescent’s eligibility for study participation with health workers or parents/primary caregivers who accompanied the adolescent.

### Data Collection

We developed a self-administered questionnaire based on the Zambia Demographic and Health Survey 2007 (33), WHO’s “HIV testing, treatment and prevention: generic tools for operational research” (34), and previous literature (14, 30, 35, 36). We collected information on background characteristics of the adolescents, the settings in which serostatus disclosures were performed, settings they preferred for serostatus disclosure, and the impacts of disclosure on their emotions and behaviors (e.g., improved/maintained adherence to ART, putting the blame on parents) (14, 30, 35). We also collected data on health education topics that the adolescents had ever learned at the hospital (e.g., benefit of adherence to ART, how to deal with emotions) (36).

Regarding the setting where disclosure took place, we asked each adolescent about his/her age at that time, the venue at which the disclosure took place, the person who disclosed HIV serostatus to him/her, whether he/she had already suspected infection with HIV, and emotional reaction upon knowing HIV serostatus. We also asked an open-ended question about why they had a particular reaction upon disclosure. We solicited their suggestions on how HIV should be disclosed, including the appropriate age, venue for disclosure, the best person to conduct the disclosure, and whether it is beneficial for adolescents to know their HIV serostatus. Furthermore, we asked an open-ended question on the perceived advantages or disadvantages of knowing HIV serostatus.

We developed the self-administered questionnaire using simple English, conducted a pre-test to assess the English literacy of the study participants, and finalized it. During the survey, trained research assistants guided or interviewed adolescents through verbal translation into local language if they had insufficient English literacy.

### Data Analysis

Out of 200 adolescents recruited, 200 were admitted to the study, 198 completed the questionnaire, and 2 withdrew during the survey. Out of 198, we included 190 in the analysis and excluded 8 as the data on their age did not meet the eligibility criteria (i.e., <15 or >19 years old) although we had asked the age of each potential participant at recruitment. We performed descriptive analysis to show the basic characteristics of the adolescents, actual settings in which they were informed about their HIV serostatus, their suggestions on how HIV disclosure should be done, emotional and behavioral impacts of disclosure, and the health education that they had ever received at the hospital.

Adolescents answered the open-ended questions with a single sentence. Based on the existing literature on qualitative data analysis (37, 38), we analyzed the open-ended answers using thematic analysis. Two authors (Sumiyo Okawa and Kimiyo Kikuchi) read and coded the textual data, and categorized all codes independently. Sumiyo Okawa and Kimiyo Kikuchi categorized the codes into sub-themes and developed main themes built on the sub-themes. Sumiyo Okawa and Kimiyo Kikuchi made a consistency check on the codes and emerging categories. This process continued until the two authors reached an agreement. All authors reviewed and finalized the emerging themes.

### Ethical Considerations

We obtained ethical approval from the Research Ethics Committee of the University of Zambia, and the Institutional Ethics Committee of the National Center for Global Health and Medicine, Japan. All adolescents provided assent to participate in the study, and parents/primary caregivers of the adolescents aged 15–17 years also offered informed consent. We collected all data anonymously.

## Results

Table 1 shows basic characteristics of the adolescents (*n* = 190). Out of them, 80 (42.1%) were boys, and 110 (57.9%) were girls. Thirty-four adolescents (17.9%) never attended school or completed basic school education; 42.1% of their primary caregivers were mothers; and at least 41.6% of the primary caregivers were living with HIV.

**Table 1:** Basic characteristics of the adolescents (*n* = 190).

Characteristics	*n* (%)
**Gender**	
Boy	80 (42.1)
Girl	110 (57.9)
**Age**	
15	28 (14.7)
16	47 (24.7)
17	39 (20.5)
18	45 (23.7)
19	31 (16.3)
**Education**	
Never educated/did not complete school	34 (17.9)
Completed basic school	93 (49.0)
Competed secondary school or higher	63 (33.2)
**Type of primary caregiver**	
Mother	80 (42.1)
Father	27 (14.2)
Grandmother	26 (13.7)
Aunt	25 (13.2)
Sister	14 (7.4)
Other (e.g., uncle, brother, cousin)	18 (9.5)
**Educational experience of primary caregiver**	
Never educated/did not complete basic school	20 (10.5)
Completed basic school	23 (12.1)
Competed secondary school or higher	128 (67.4)
Don’t know	19 (10.0)
**HIV status of primary caregiver**	
Positive	79 (41.6)
Negative	85 (44.7)
Don’t know	26 (13.7)

Table 2 presents the adolescents’ actual experience and suggestions for disclosure of HIV serostatus. Median age at HIV serostatus disclosure was age 12 (interquartile range 11–15)—close to the survey participants’ common suggestion on the appropriate age for disclosure 12 (interquartile range 10–14). They reported that their status was disclosed at a health facility (55.3%) or at home (37.9%). Their HIV serostatus was disclosed to them by their mothers (29.5%), health workers (27.4%), their father (7.9%), and both parents (6.3%). Majority (62.6%) suggested that having both parents disclose to their adolescent would be preferable. Over 31% had already suspected they had HIV before disclosure of their status. Upon disclosure, 45.3% had negative feelings, 27.9% had mixed feelings, while only 7.9% had positive feelings. However, 86.2% of adolescents perceived self-awareness of HIV serostatus as beneficial.

**Table 2:** Actual experience and suggestions for HIV serostatus disclosure to adolescents.

	Actual experience	Suggestion
	
	*n* (%)	*n* (%)
**Place of first knowing your HIV serostatus**		
Clinic/hospital	105 (55.3)	102 (53.7)
Home	72 (37.9)	78 (41.1)
Other	13 (6.8)	10 (5.3)
**Person who disclosed your HIV serostatus**		
Both parents	12 (6.3)	119 (62.6)
Mother only	56 (29.5)	21 (11.1)
Father only	15 (7.9)	2 (1.1)
Health workers	52 (27.4)	32 (16.8)
Other (e.g., grandparents, aunt, uncle, sibling)	50 (26.3)	15 (7.9)
Don’t remember	5 (2.6)	1 (0.5)
**Suspected HIV-positive status before disclosure**		
Yes	60 (31.6)	
No	117 (61.6)	
Don’t remember	13 (6.8)	
**Emotional reaction at first knowing HIV serostatus**		
Positive feeling	15 (7.9)	
Negative feeling	86 (45.3)	
Mixed feeling	53 (27.9)	
No feeling	17 (9.0)	
Don’t remember	19 (10.0)	
**Perception of self-awareness of HIV serostatus at the time of survey^a^**		
Beneficial		163 (86.2)
Not beneficial		19 (10.1)
Don’t know		7 (3.7)

*^a^189 included, and 1 declined to answer*.

Table 3 shows the six emerging themes regarding the adolescents’ first impressions upon disclosure of their HIV serostatus: (1) awareness/readiness (2), emotional reaction (3), existential perspective, (4) self-image, (5) perception of HIV and medication, and (6) stigma. A majority of the adolescents had negative impressions, as they never expected to be infected with HIV. One adolescent expressed, *“I never suspected I could have HIV and the worst part was that it came from my mother” (Girl, age 19)*. They were emotionally shocked, and some desired to commit suicide. Moreover, they were anxious about dying soon, had a sense of isolation as if being the only person living with HIV, and had fear of being stigmatized: *“I felt as though my life has crumbled and that I now have a burden to carry, and eventually leading to death” (Boy, age 19)*. They perceived HIV as an incurable disease and felt difficulty in daily medication for the rest of their life: *“I felt low because I would have to take my medicine for life” (Boy, age 16)*.

**Table 3:** First impression upon disclosure of HIV serostatus.

Major themes	Sub-themes	

	Positive impression	Negative impression
Awareness/readiness	Already aware of HIV serostatus	Never expected
		Not ready to accept HIV serostatus

Emotional reaction	Happy to know HIV serostatus	Shocked
		Desire to commit suicide

Existential perspective	Able to live as normal	Anxious about dying soon
	Able to live positively	Unable to have a normal life anymore
	Able to live longer	

Self-image	I am not the only person living with HIV	I am the only person living with HIV

Perception of HIV and medication	Trust in effectiveness of medicine	HIV is incurable or a bad disease
		Don’t want to take medicine daily/for rest of life
		Concern about dependency on medicine

Stigma	–	Anticipated stigma

On the other hand, some adolescents showed a positive impression as they had already suspected being infected with HIV, or they believed that the medicine should work well and that they could live as long as or even longer than other people: *“I felt good because I knew my status and I was able to live positive life” (Girl, age 17)*, and *“I felt sicker when I knew about my status, so I started taking ARVs. I became healthier than ever and happy” (Girl, age 19)*.

Table 4 shows the impact of disclosure of HIV serostatus on the emotions and behaviors of adolescents. Majority (84.7%) improved or did not change adherence to ART. Regarding relationships with parents or primary caregivers, as a consequence of disclosure 54.2% felt more comfortable talking about HIV with their parents or caregivers, while 32.1% blamed their parents. Eighty-one percent limited self-disclosure of their HIV serostatus to others, and 31.1% felt stressed about keeping the status a secret. In relationships with friends or intimate partners, 42.5% became scared of developing intimate relationships, and 12.1% isolated themselves from friends.

**Table 4:** Impact of disclosure of HIV serostatus on adolescents’ emotion and behavior (*n* = 190).

Impacts	*n* (%)
**Physical health**	
Improved/kept adherence to antiretroviral therapy (ART)^a^	149 (84.7)
Worried about my health	136 (71.6)
**Relationship with parents/caregivers**	
Felt comfortable to talk about HIV with caregiver	103 (54.2)
Blamed my parents	61 (32.1)
**Disclosing HIV status to other**	
Limited self-disclosure to others	154 (81.1)
Felt stressed to keep my HIV status secret	59 (31.1)
**Relationship with friends and intimate partners**	
Felt scared to develop an intimate relationship^b^	59 (42.5)
Stopped sexual relationship^c^	21 (32.3)
Ever felt bad because someone talked about my HIV status to others	34 (17.9)
Isolated myself from my friends	23 (12.1)

*^a^Among those who have initiated ART (*n* = 176)*.

*^b^Among those who have had an intimate partner (*n* = 139)*.

*^c^Among those who have engaged in sexual relationship (*n* = 65)*.

Table 5 shows perceived advantages and disadvantages of HIV serostatus disclosure. Majority believed that it is important to know the reasons for being sick and purpose of taking medicine. Some adolescents regarded knowing one’s own HIV serostatus as their basic right. Being aware of their HIV serostatus also helps adolescents to enhance self-care behavior, including adherence to ART, improvement in their quality of life, and prevention of HIV transmission to others. For example, an adolescent mentioned that *“Knowing HIV status helps them to move to another level in life” (Boy, age 16)*. On the other hand, they were concerned that some adolescents might not be mature enough to accept their HIV serostatus, become emotionally distressed, unnecessarily disclose their HIV serostatus to other people, and be discouraged about taking medicine. An adolescent expressed, *“Some would feel as though it is the end of the world, and some would not know how to handle their feelings” (Boy, age 19)*.

**Table 5:** Perceived advantages and disadvantages of disclosing HIV serostatus to adolescents.

Perception	Detail
Advantages	Improve self-care
	Improve adherence to medicine
	Improve quality of life
	Knowing HIV status is beneficial/human right
	Knowing the objective for taking medicine is beneficial
	Able to accept HIV status
	Prevent HIV transmission to others

Disadvantages	Cause emotional distress
	Disclose HIV status to others unnecessarily
	Not mature to accept HIV status
	Discourage to take medicine

Neutral	Depends on one’s personality or characteristics

Table 6 shows health education topics that adolescents had ever learned at the hospital. Regarding the topics about HIV and treatment, 89.0% had learned about the benefits of adherence to ART, 77.4% had learned about the risks of non-adherence to ART, and 75.8% had learned about the duration of taking ART once it begins. Seventy-six percent had learned about general modes of HIV transmission, while 69.5% had heard about how they got HIV infection. Regarding the topics about psychosocial well-being, a lower proportion of adolescents had learned about how to deal with their emotions (63.2%), compared with those who had learned about how to develop hope for the future (79.5%) and how to develop self-esteem (69.0%).

**Table 6:** Health education topics that adolescents had ever learned at hospital (*n* = 190).

Topics	*n* (%)
**HIV and treatment**	
Benefit of adherence to antiretroviral therapy (ART)	169 (89.0)
Risk of non-adherence to ART	147 (77.4)
General mode of HIV transmission	145 (76.3)
Duration of taking ART once it starts	144 (75.8)
How I got HIV infection	132 (69.5)
**Psychosocial well-being**	
How to develop hope for the future	151 (79.5)
How to develop self-esteem	131 (69.0)
How to deal with emotions	120 (63.2)

## Discussion

This study examined disclosure of HIV serostatus to adolescents living with HIV in Zambia using quantitative data and developing triangulate evidence from existing qualitative findings (14, 22, 30). Particularly, we probed whether parents and caregivers’ concerns before disclosing HIV serostatus to adolescents such as psychologically traumatizing adolescents, being blamed for HIV transmission, and risk of exposing adolescents to stigma in community (22) eventually materialized. We also identified the appropriate setting for disclosing HIV serostatus based on the adolescents’ reported experiences.

High proportion of the adolescents developed negative feelings (45.3%) or mixed feelings (27.9%) when they were informed of their HIV serostatus, and 71.6% became worried about their health. This is because HIV infection was unexpected and perceived to be an incurable and fatal disease. However, majority of the adolescents (86.2%) reported that knowing their HIV serostatus is beneficial. Over 84% of the adolescents reported improved adherence to ART after serostatus disclosure. Disclosure helped them to understand the reasons for being sick and the purpose for taking medicine, enhanced motivation to care for one’s own health, and improved quality of life. Similarly, in Zimbabwe and South Africa, adolescents showed extreme shock immediately after their HIV-positive status was disclosed, but they understood that knowing one’s own HIV status is important for adherence to ARVs and to stay alive (10, 18). This implies that despite initial distress, they had resilience to accept and live with HIV. However, fear of inflicting emotional distress is a major barrier for parents and primary caregivers to disclose HIV serostatus to adolescents (22). Therefore, the important strategy will be to arrange both pre- and post-disclosure care and support for adolescents and their parents/caregivers until the adolescents can accept their HIV serostatus sufficiently.

The study also showed adolescents’ reactions upon serostatus disclosure that usually pose concerns among parents/primary caregivers. In previous studies, parents were reluctant to disclose HIV serostatus to their adolescents due to fears of losing reputation and being blamed by them for transmitting the virus (14, 22). However, more than half of the adolescents in this study (54.2%) reported better communication with their parents/caregivers when discussing HIV; with a lower percentage of those blaming their parents (32.1%). Similarly, in Kenya, parents/caregivers of HIV-positive adolescents experienced improved relationships with their adolescents (39).

Parents/primary caregivers were also concerned about exposing their adolescents to stigma if their HIV serostatus was disclosed to others (22). However, 81.1% of the adolescents in this study did not self-disclose to others; 31.1% felt stressed about keeping HIV serostatus in secret; and only 17.9% had ever felt bad about disclosure by someone without their permission. Similarly, in South Africa, adolescents understood the secret nature of their HIV serostatus to avoid gossip and stigma, particularly when their parents/caregivers instructed them not to disclose to others (10). This indicated that parents/caregivers benefit from disclosing HIV serostatus to adolescents. They need to talk to their adolescents about positive and negative consequences of self-disclosing their HIV serostatus, and trust their ability to keep matters private. In addition, health workers should take care of psychological well-being of adolescents and provide counseling to parents/caregivers when they encounter difficult relationships with their adolescents after disclosure.

To improve the practice of disclosure of HIV serostatus, it is worth comparing adolescents’ actual experiences with what they perceive as appropriate. The adolescents were disclosed their HIV serostatus at median age of 12 years; they regarded such age as appropriate for other adolescents too. This corresponds with the WHO recommendation (27). Although caregivers considered maturity of an adolescent as an important criterion for disclosure in Zambia (22), our study finding encourages parents/primary caregivers to prepare for disclosure of HIV serostatus to their adolescents before they turn 12 years old.

Disclosure was usually done at the hospital or adolescent’s home, which was similar to the adolescents’ suggestions. However, only 6% of the adolescents were actually informed by both parents, while over 60% preferred disclosure by both parents. Existing literature shows that adolescents and their parents/caregivers preferred disclosure by caregivers with assistance from health-care workers as they have accurate knowledge about HIV (25). Thus, disclosure by both parents is the preferred setting for Zambian adolescents, and additional assistance of health workers would be helpful to facilitate emotional and intellectual acceptance of HIV serostatus, although most adolescents have lost one or both parents due to HIV/AIDS.

Learning about HIV and its implications for adolescents’ daily life is a critical part of care associated with disclosing HIV serostatus. At the hospital, adolescents acquired basic knowledge about HIV (e.g., mode of HIV transmission, benefits of adherence, and risk of non-adherence to ART). On the other hand, they did not learn much about psychosocial well-being (i.e., self-esteem development and emotional adjustment). They need support for developing self-esteem and mitigating emotional distress. This is because, even though they do not disclose their HIV serostatus to others, their serostatus could be spread out, and they may encounter social exclusion. Professional care and counseling are essential to respond to their psychosocial needs (30). In addition, peer support can also provide knowledge about HIV, mitigate emotional distress, and empower each other (14, 18). About 8% of the adolescents in this study reported a positive acceptance of their HIV serostatus immediately after disclosure. A recommendation for a future study is to investigate factors that enabled these adolescents to accept their HIV serostatus with minimal distress, as this information could be adopted in the pre- and post-disclosure counseling and peer support activity.

Recommendations to health workers are to provide information on benefits and potential risks of disclosure to caregivers, including the study finding that their concerns before disclosure do not necessarily materialize. The information will mitigate caregivers’ anxiety and facilitate their preparation for disclosure. Disclosing HIV status is a significant life event for majority of the adolescents. Health workers should pay attention to emotional impact of adolescents upon disclosure. Moreover, health workers need to perform counseling and educating adolescents on a wider range of topics including psychosocial well-being, involving trained peer supporters.

This study has several limitations. The study design may limit generalizability of the results as the study was conducted in one tertiary hospital in the capital city of Zambia. Recruiting adolescents at their regular clinical reviews may also limit generalizability of the findings as those who were not compliant with care instructions or had no access to care were excluded from the study; those adolescents may have different views or different impacts of disclosure. A recommendation for the future study is to conduct a longitudinal study design to assess process, and emotional and behavioral impacts of serostatus disclosure to adolescents, including rural settings where local norms and HIV care and treatment services for adolescents would be different from an urban setting. Another limitation is that adolescents may have felt obligated to participate in the study as the study site was the place where they were receiving the care, and the participants may not have been mature enough to decline to participate. We carefully explained to each adolescent at recruitment about voluntary participation and freedom of withdrawal, with no negative implications for health-care they would receive. Moreover, the accuracy of the study results might be affected by recall bias because of the time gap between the disclosure event and participation in the survey.

## Conclusion

This study strengthens existing evidence on HIV serostatus disclosure to adolescents in Zambia. Disclosing HIV serostatus to adolescents has a strong impact on their emotions. However, it improved self-care and adherence to ART. In contrast to parents/primary caregivers’ concerns, disclosing HIV serostatus to adolescents also promoted better communication, while it was not a trigger for unnecessary self-disclosure to others. They prefer to be informed of their HIV serostatus by their parents at the age of 12. Serostatus disclosure to adolescents requires continuous care with the commitment of parents/primary caregivers, health workers, and peer adolescents.

## Ethics Statement

We obtained ethical approval from the Research Ethics Committee of the University of Zambia, and the Institutional Ethics Committee of the National Center for Global Health and Medicine, Japan. All adolescents provided assent to participate in the study, and parents/primary caregivers of the adolescents aged 15–17 years also offered informed consent. We collected all data anonymously.

## Author Contributions

SO, NI, SM-K, MM, and CK substantially contributed to the conception and design of the work, and the acquisition. SO, NI, SM-K, and KK contributed to the analysis and interpretation of data for the work. SO, NI, SM-K, and MJ drafted the work. All authors revised it critically for important intellectual content, made final approval of the version to be published, and agreed to be accountable for all aspects of the work in ensuring that questions related to the accuracy or integrity of any part of the work are appropriately investigated and resolved.

## Conflict of Interest Statement

The authors declare that the research was conducted in the absence of any commercial or financial relationships that could be construed as a potential conflict of interest.
